# Dental MRI of Oral Soft-Tissue Tumors—Optimized Use of Black Bone MRI Sequences and a 15-Channel Mandibular Coil

**DOI:** 10.3390/jimaging8050146

**Published:** 2022-05-22

**Authors:** Adib Al-Haj Husain, Esra Sekerci, Daphne Schönegg, Fabienne A. Bosshard, Bernd Stadlinger, Sebastian Winklhofer, Marco Piccirelli, Silvio Valdec

**Affiliations:** 1 Clinic of Cranio-Maxillofacial and Oral Surgery, Center of Dental Medicine, University of Zurich, 8032 Zurich, Switzerland; adib.al-hajhusain@uzh.ch (A.A.-H.H.); esra.sekerci@zzm.uzh.ch (E.S.); fabienne.bosshard@zzm.uzh.ch (F.A.B.); bernd.stadlinger@zzm.uzh.ch (B.S.); 2 Department of Cranio-Maxillo-Facial and Oral Surgery, University Hospital of Zurich, University of Zurich, 8091 Zurich, Switzerland; daphne.schoenegg@usz.ch; 3 Department of Neuroradiology, Clinical Neuroscience Center, University Hospital of Zurich, University of Zurich, 8091 Zurich, Switzerland; sebastian.winklhofer@usz.ch (S.W.); marco.piccirelli@usz.ch (M.P.)

**Keywords:** oral soft-tissue tumors, irritation fibroma, magnetic resonance imaging, dental MRI, mandibular coil, hyperplasia, reactive lesions, oral radiology, oral and maxillofacial surgery

## Abstract

Soft-tissue lesions in the oral cavity, one of the most common sites for tumors and tumor-like lesions, can be challenging to diagnose and treat due to the wide spectrum from benign indolent to invasive malignant lesions. We report an abnormally large, rapidly growing hyperplastic lesion originating from the buccal mucosa in a 28-year-old male patient. Clinical examination revealed a well-circumscribed, smooth-surfaced, pinkish nodular lesion measuring 2.3 × 2 cm, which suggested the differential diagnosis of irritation fibroma, pyogenic granuloma, oral lipoma, and other benign or malignant neoplasms such as hemangioma, non-Hodgkin’s lymphoma, or metastases to the oral cavity. Dental MRI using a 15-channel mandibular coil was performed to improve perioperative radiological and surgical management, avoiding adverse intraoperative events and misdiagnosis of vascular malformations, especially hemangiomas. Black bone MRI protocols such as STIR (short-tau inversion recovery) and DESS (double-echo steady-state) were used for high-resolution radiation-free imaging. Radiologic findings supported the suspected diagnosis of an irritation fibroma and ruled out any further head and neck lesions, therefore complete surgical resection was performed. Histology confirmed the tentative diagnosis. This article evaluates the use of this novel technique for MR diagnosis in the perioperative management of soft-tissue tumors in oral and maxillofacial surgery.

## 1. Introduction

The oral cavity is considered one of the most common sites for tumors and tumor-like lesions, which may be odontogenic or nonodontogenic in origin. These oral mucosa disorders can be difficult to diagnose and treat, as they include a broad spectrum from benign indolent to invasive malignant lesions of various etiologies. Many of these oral soft-tissue lesions tend to become chronic and affect the patient’s quality of life [1,2,3].

Oral fibromas, benign lesions originating from connective tissue, are the most common tumors of the oral cavity [4]. In daily clinical routine, it can be challenging to distinguish between true neoplasms and reactive fibroconnective tissue hyperplasias. Most oral irritation fibromas develop mainly in response to chronic local irritation or trauma; however, the precise etiology is not yet fully understood. The leading causes of mechanical irritation are habitual parafunctional biting on the mucosa or iatrogenic factors such as an overhanging or fractured dental restoration or an improperly fitting prosthesis [5,6]. The most common clinical presentation is a slowly and painlessly growing well-circumscribed, smooth-surfaced, normally colored mucosal lesion of hard consistency [7]. The typically pedunculated solitary lesion is usually smaller than 1.5 cm, although single rare cases of a larger size are described in the literature [7,8]. It affects patients between the third and sixth decade of life and occurs in 1.2% of adults, predominantly women (66%) [9]. Most irritation fibromas are localized on the labial mucosa, gingiva, and the tip of the tongue, causing difficulties in mastication and speech [10]. Differential diagnosis includes pyogenic granuloma, lipoma, and other benign or malignant neoplasms such as hemangiomas, non-Hodgkin’s lymphoma, or metastasis in the oral cavity [9]. Therefore, a thorough multidisciplinary coordinated preoperative evaluation of all case-specific factors, including medical history, clinical, and especially radiologic and histopathologic features, is key in borderline cases to avoid inappropriate treatment.

Recent developments in dentomaxillofacial imaging and state-of-the-art computer technologies reveal that the basis for oral and maxillofacial radiological assessment and preoperative surgical planning is constantly evolving. Thus, cross-sectional imaging is considered an advanced and indispensable support tool in modern medical diagnostics that complements the clinical examination [11]. While conventional X-ray-based imaging modalities such as panoramic radiography (PAN) or cone-beam computed tomography (CBCT) are considered the “gold standard” for hard tissue visualization, they are limited for soft-tissue imaging. Magnetic resonance imaging (MRI), which is firmly established for diagnosis in the head and neck region, is ideally suited and superior for soft-tissue visualization [11]. Compared to conventional radiological imaging techniques, it offers the advantage of visualizing complex anatomical changes in soft-tissue pathologies in the oral cavity without exposure to X-rays [12].

MRI has evolved rapidly over the past decades with various technical novelties and advanced imaging protocols, providing a broad spectrum of new diagnostic capabilities for the dental field [13]. Dental MRI with the recently introduced black bone MRI sequences is another step towards simultaneous radiation-free imaging of soft and hard tissues in the oral and maxillofacial region [14,15,16,17]. It allows for better characterization and staging of benign and malignant oral changes, providing the clinician with surgically relevant information such as the exact depth extent of clinically suspicious lesions, or information on other, clinically unsuspected, changes [18,19,20,21]. To overcome the remaining limitations of MRI in the oral cavity, such as motion artifacts and artifacts due to field inhomogeneities caused by metallic reconstructions [22], indication-specific MRI protocols in combination with novel imaging tools, such as radiofrequency coils [23], wireless intraoral coils [24] or mandibular coils [25], are implemented in the image acquisition process to achieve better image quality with shorter acquisition times.

This article reports an abnormally large hyperplastic lesion originating from the buccal mucosa of a 28-year-old male patient with relatively rapid growth and displacement of the maxillary second molar. Dental MRI with a 15-channel mandibular coil was performed to improve perioperative management. Specific black bone MRI protocols such as STIR (short-tau inversion recovery) and DESS (double-echo steady-state) were used to image soft-tissue lesions in the oral cavity with high resolution. In addition, the current literature is reviewed and evaluated for evidence-based perioperative clinical and radiological case management.

## 2. Material and Methods

### 2.1. Case Presentation

A 28-year-old male patient presented to the Clinic of Cranio-Maxillofacial and Oral Surgery, Center of Dental Medicine, University of Zurich (Switzerland), complaining of pain and swelling of the left cheek. He reported rapid, uncomfortable growth of a lesion originating from the buccal mucosa over the past 6 months. He had initially noticed the lesion three years earlier after removing braces. Recently, the lesion started to occasionally impair his masticatory function, causing him to bite himself accidentally.

The dental history revealed no abnormalities or evidence of infection or trauma in the affected region. The patient’s medical history was unremarkable.

**Figure 1:**
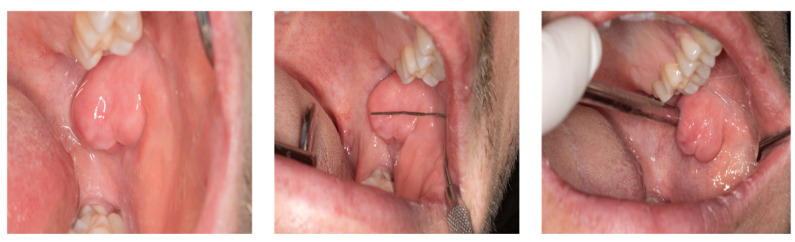
Intraoral visualization of an abnormally large hyperplastic lesion originating from the buccal mucosa in a 28-year-old patient presenting to the Department of Oral and Maxillofacial Surgery. Besides a relatively rapid growth of lesion size over the past six months, a displacement of the maxillary second molar could be observed. In addition to the main complaints of pain and swelling, clinical examination revealed a well-circumscribed, smooth-surfaced, pinkish nodular swelling measuring 23 × 20 mm in the left planum buccale.

Intraoral clinical examination revealed a well-circumscribed, smooth-surfaced mobile, nodular swelling measuring approximately 2.3 × 2 cm in the left planum buccale, covered with normal pink mucosa. The lesion was hard and noncompressible on palpation, the temperature was not elevated, and the lymph nodes were neither enlarged nor palpable. In addition, no increased teeth mobility was observed in the affected region (Figure 1).

### 2.2. Dental Magnetic Resonance Imaging—Data Acquisition

**Figure 2:**
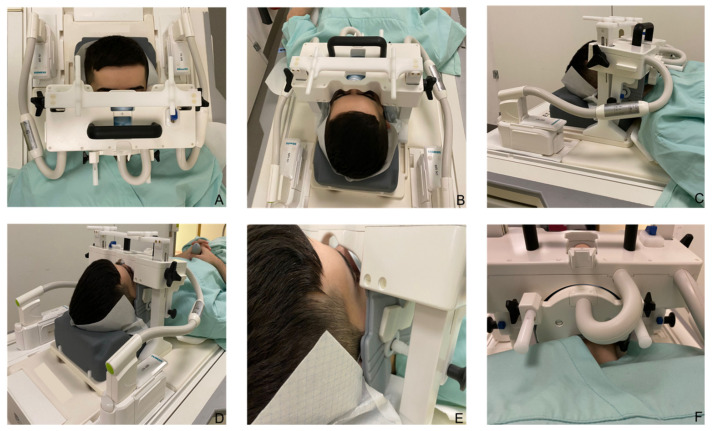
The 15-channel mandibular coil (NORAS MRI products, Hoechberg, Germany) is presented. (**A**–**C**) The mandibular coil used in this setup is an optimized 14 + 1 receive coil array and position system specifically designed for high-resolution imaging of dental structures in the oral cavity. It compromises a curved 12 × 38 cm^2^, 14 elements phased array coil between two bars. (**D**) Fixation elements allow precise positioning of the patient’s head in the anteroposterior and cranio-caudal direction. (**E**) The outer wings of the array coil are flexible and can be freely and precisely adapted to the patient’s individual jaw anatomy. (**F**) A mirror and head fixation can be attached to increase the patient’s comfort, minimize motion artifacts, and reduce distress for claustrophobic patients.

Based on the clinical presentation, the differential diagnosis included pyogenic granuloma, oral lipoma, and other benign or malignant neoplasms such as hemangioma, non-Hodgkin’s lymphoma, or metastasis in the oral cavity. Therefore, to improve perioperative radiological and surgical management, dental MRI was performed using a 15-channel mandibular coil (NORAS MRI products, Hoechberg, Germany) to provide high spatial resolution and excellent soft-tissue contrast (Figure 2).

The 15-channel mandibular coil (field of view: 32 × 16 × 16 cm) used in this setup is an optimized 14 + 1 receive coil array and position system specifically designed for high-resolution imaging of dental structures in the oral cavity. It compromises a curved 12 × 38 cm^2^, 14 elements phased array coil between two bars. Fasteners allow precise positioning of the patient’s head in the anteroposterior and cranio-caudal direction. As visualized in Figure 2, there are openings for the nose and mouth. The central junction between the two openings should be positioned directly above the upper lip. The outer wings of the array coil are flexible and can be freely and precisely adapted to the patient’s individual jaw anatomy. Patients do not have to open their mouth or bite down on anything in any particular position when using the mandibular coil. Additionally, a mirror and head fixation can be attached to increase the patient’s comfort, minimize motion artifacts, and reduce distress for claustrophobic patients. The multielement receive array and positioning system facilitates faster imaging data acquisition by parallel imaging and subsequent k-space undersampling [25].

Dental magnetic resonance imaging protocols included conventional MRI protocols; native T1 ax and T2 fat-saturated (FS) cor Turbo Spin-Echo (TSE) 2D acquisitions, a 3D DESS protocol, a 3D-T2-STIR protocol, and a T2-weighted Dixon TSE protocol. MR images were acquired on a 3-Tesla Skyra (release VE11e, Siemens Healthineers, Erlangen, Germany) with the following main technical scanning parameters:

DESS with water excitation protocol had an isotropic acquisition resolution of 0.75 × 0.75 × 0.75 mm^3^ together with a receive bandwidth of 355 Hz/Px. The other sequence parameters were: field of view, 242 × 242 × 78 mm^3^; acquisition matrix, 320 × 320 × 104; slice oversampling, 100%; no parallel acquisition; one signal average; acquisition time, 12:24 min:s; TR/TE1/TE2 11.2/4.2/7.7 ms; flip angle, 30°; selective water excitation; 2D T1 TSE ax: field of view, 180 × 180 × 99 mm^3^; acquisition matrix, 384 × 288; 30 slices; acq. resolution of 0.47 × 0.62 × 3.0 mm^3^; receive bandwidth of 260 Hz/Px.; TR/TE 482/9.5 ms; flip angle, 134°; phase oversampling, 60%; echo trains per slice/TSE factor, 154/3; no parallel acquisition; 1 signal average; acquisition time, 3:46 min:s; 2D T2 TSE FS ax: field of view, 180 × 180 × 86 mm^3^; acquisition matrix, 384 × 307; 26 slices; acq. resolution of 0.47 × 0.59 × 3.0 mm^3^; receive bandwidth of 260 Hz/Px.; TR/TE 7710/89 ms; flip angle, 150°; fatsat; phase oversampling, 10%; echo trains per slice/TSE factor, 20/17; no parallel acquisition; 2 signal averages; acquisition time, 5:25 min:s; 3D T2 TSE STIR cor: field of view, 210 × 210 × 64 mm^3^; acquisition matrix, 256 × 243 × 64; acq. resolution of 0.82 × 0.86 × 1.0 mm^3^; receive bandwidth of 630 Hz/Px. TR/TE/TI 3300/197/220 ms; flip angle mode, T2 var; phase/slice oversampling, 70/12.5%; phase, GRAPPA 2; 1.4 signal average; acquisition time, 7:04 min:s; 2D T2 TSE Dixon ax: field of view, 210 × 210 × 168 mm^3^; acquisition matrix, 320 × 240; 43 slices; acq. resolution of 0.66 × 0.88 × 3.0 mm^3^; receive bandwidth of 340 Hz/Px.; TR/TE 5000/80 ms; flip angle, 131°; phase oversampling, 60%; echo trains per slice/TSE factor, 11/19; GRAPPA 2; 1 signal averages; acquisition time, 3:52 min:s (Table 1).

**Figure 3:**
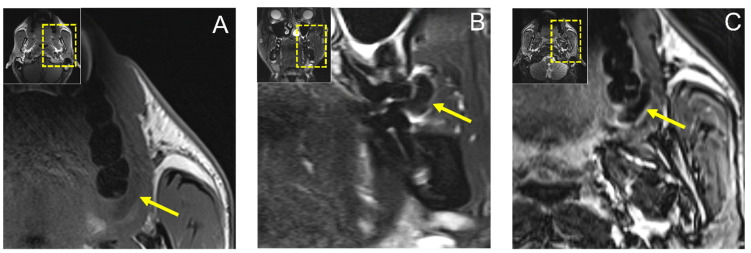
(**A**) Axial reconstruction of native T1 and (**B**) coronal reconstruction of T2 fat-saturated (FS) Turbo Spin-Echo (TSE) MRI protocols showing a hypointense lesion in T1-weighted images in axial orientation measuring approximately 20 × 5 mm and a 12 × 8 mm hypointense lesion in coronal T2 FS TSE reconstruction, ruling out a fat-containing tumor or cystic process. In addition, (**C**) axial T2-weighted Dixon TSE reconstruction is shown.

MR imaging with conventional noncontrast T1 and T2 fat-saturated (FS) turbo spin-echo (TSE) protocols revealed a hypointense lesion in T1-weighted images in the axial orientation measuring approximately 20 × 5 mm and a 12 × 8 mm hypointense lesion in the coronal T2 FS TSE reconstruction, ruling out a fat-containing tumor or cystic process (Figure 3).

**Figure 4:**
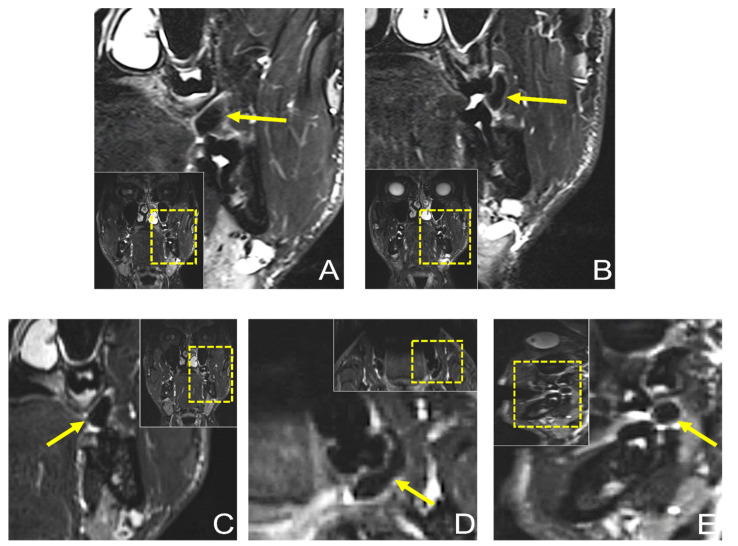
Preoperative dental MRI showed a well-demarcated lesion with a maximum extension of (**D**) 21.3 × 5.6 mm (axial), (**A**–**C**) 13 × 8.6 mm (coronal), and (**E**) 10.1 × 9.8 mm (sagittal) on a T2 (short-tau inversion recovery) STIR protocol, with homogenous low signal intensity in the central area of the lesion, while the peripheral area showed a high signal intensity. The lesion originated from the planum buccale and did not infiltrate adjacent structures, with (**E**) a displacement of the second upper molar. For orientation, the dotted rectangles in the corner show the enlarged area.

**Figure 5:**
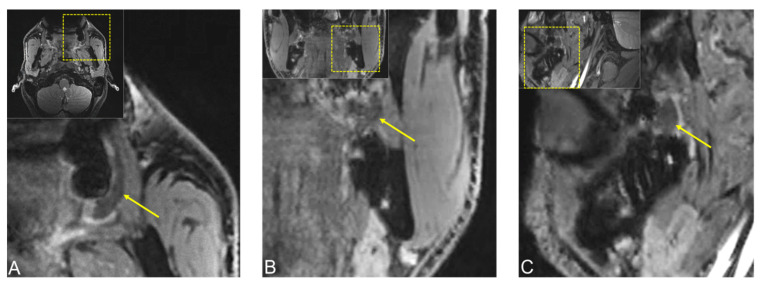
Preoperative MRI of the same lesion using 3D double-echo steady-state (3D-DESS) imaging protocol. (**A**) Axial, (**B**) coronal, and (**C**) sagittal reconstructions visualizing a well-demarcated lesion with a maximum extension of 21.3 × 5.6 mm (axial), 13 × 8.6 mm (coronal), and 10.1 × 9.8 mm (sagittal). For orientation, the dotted rectangles in the corner show the enlarged area. The MR image reconstructions seem to have a slightly lower resolution due to the larger scale along the slice direction.

Dental MRI showed a well-demarcated lesion with a maximum extension of 21.3 × 5.6 mm (axial), 13 × 8.6 mm (coronal), and 10.1 × 9.8 mm (sagittal) on a T2 STIR protocol, with homogenous low signal intensity in the central area of the lesion, while the peripheral area showed a high signal intensity. The lesion originated from the planum buccale and did not infiltrate adjacent structures, with a displacement of the second upper molar (Figure 4). The DESS protocol revealed the same hypointense lesion with lower resolution and image quality compared with the STIR protocol (Figure 5). Thus, the radiological findings excluded any further involvement in the head and neck area, the presence of a lipoma, or other space-occupying pathologies, and provided the precise localization and extension of the suspected fibromatous lesion.

### 2.3. Surgery and Histopathology

**Figure 6:**
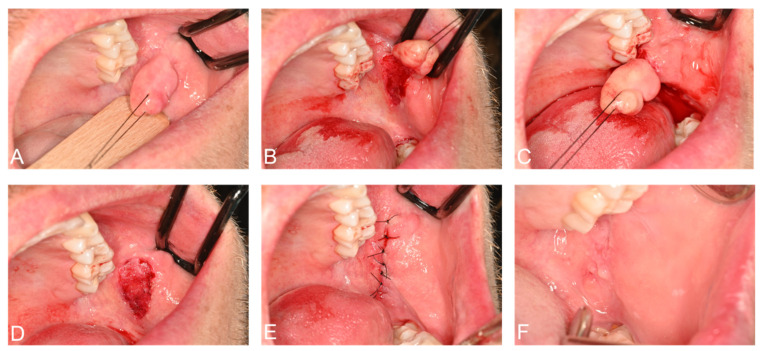
(**A–E**) Complete surgical excision of the irritation fibroma was performed under local anesthesia without perioperative complications. The lesion was sent for histological examination. (**F**) Postoperative situation one week after surgery, when sutures were removed, showed adequate wound healing.

**Figure 7:**
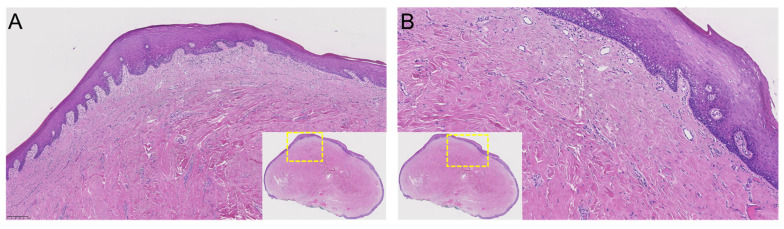
(**A,B**) Histopathological features of the specimen (**A**), original magnification ×5, and (**B**) original magnification ×10).

Thus, complete surgical excision of the lesion was planned. The surgical procedure was performed under local anesthesia and aseptic conditions without perioperative complications. Safety precautions included wearing goggles, using gauze in the surgical field, and suctioning under high vacuum. The lesion was completely excised with a BP blade (no. 15). After good coagulation, the wound was sutured (PTFE 3-0). The patient was prescribed chlorhexidine mouth rinse two times a day for 5 days (Figure 6). The lesion was sent for histological examination. Histopathological examination revealed a polypoid mucosal excision with an intact epithelium and no dysplasia, with no evidence of malignancy or significant inflammatory infiltrates. The fibrous connective tissue, which was dense and showed a circular collagenized pattern, was covered by stratified squamous epithelium. Periodic acid–Schiff (PAS) staining revealed no evidence of fungal hyphae. Based on these microscopic findings, the diagnosis of irritation fibroma was confirmed (Figure 7). Close postoperative clinical follow-up of the patient is scheduled, with monthly check-ups during the first year. Subsequently, the lesion should be checked as part of the annual dental check-up, whereby the patient can present again at any time if necessary.

## 3. Discussion

Soft-tissue lesions of the mucosa can be diverse and difficult to diagnose and treat, as there can be multiple predisposing factors for tumor development, such as poor oral hygiene, excessive smoking, harmful habits, insufficient dental restorations, or mechanical irritation. The proven initial tools for diagnosis are clinical and radiological findings, which are important for early detection of any premalignant respective malignant pathologic abnormalities, although the definitive diagnosis is provided on the basis of histopathological analysis [3]. Despite the importance of reliable diagnosis, some soft tissue lesions in the oral cavity are difficult to biopsy due to the complex anatomical courses, small size, and variations of the nerves and blood vessels. Therefore, there is always a risk of injuring adjacent structures such as tooth roots, nerves, blood vessels, or salivary gland ducts in high-risk cases, necessitating more conservative diagnostic options such as radiation-free imaging techniques that provide excellent soft tissue contrast.

Oral irritation fibromas, a usually focal proliferation of fibrous tissue secondary to chronic irritation, can be identified in most cases with a relatively high degree of confidence based on medical history and clinical features. However, differentiation can be difficult, as it can have a similar clinical appearance to other benign or malignant neoplasms [4]. In the presented case, many clinical features supported the tentative diagnosis, e.g., localization, solitary lesion, well-circumscribed nodule, yet the relatively rapid growth over the past six months and unusually large size warranted a multimodality preoperative diagnostic approach.

Histopathological examination is also the most effective diagnostic tool for diagnosing irritation fibromas with the most significant possible degree of certainty. As for etiology, the precise mechanisms of tissue enlargement are still unclear, but irritant fibromas show a specific pattern of collagen fiber arrangement that depends on the location of the lesion and the extent and direction of chronic irritation. There are two types of patterns: the radiating pattern, which occurs in immobile sites with a higher degree of trauma, such as the palate, and the circular pattern, which appears in more flexible anatomic regions, such as the buccal mucosa due to minor trauma [6]. It is suspected that other factors, such as plaque microorganisms, might also be co-factors contributing to the pathogenesis [26]. Irritation fibromas often reveal an unencapsulated nodular mass of fibrous connective tissue with large fibroblasts covered by squamous epithelium. The collagen patterns observed may be radiating, circular, or irregular. In addition, epithelial atrophy or hyperkeratosis may be observed due to secondary trauma to the surface [27]. Treatment of an irritation fibroma should always consist, at a minimum, of eliminating iatrogenic factors, which is critical to the success of any selected form of therapy [27]. This conservative treatment option has the potential to eliminate or at least reduce the size of the lesion. However, radical surgical resection is the best treatment option to minimize the risk of recurrence. Long-term postoperative follow-up is essential because of the high growth potential of the incompletely excised lesion. Recurrence after complete excision on the other hand is rare [28]. 

In addition to established diagnostic tools, advances in dental MRI with the use of black bone MRI sequences such as STIR or DESS open the possibility of detailed anatomical images of soft and hard tissues in the oral and maxillofacial region, leading to improved preoperative radiological assessment and surgical planning [14,15,29,30]. Older MRI studies assessing visualization of the oral cavity used conventional, nonspecific MRI protocols with a magnetic field strength of 1 Tesla, resulting in a low signal-to-noise ratio. Compared to the current standard, the images obtained provided insufficient image resolution for clinical use. The use of higher field strengths of 3 Tesla and coils designed explicitly for dental imaging, such as radiofrequency coils [23], wireless intraoral coils [24], or mandibular coils [25], enabled excellent image quality with a high spatial resolution of the alveolar ridge and teeth with the surrounding soft-tissues [31]. Refinement of the MR sequences using ultrashort echo times was successfully implemented in various dental tasks [19,21,30,32]. Recently, the implementation of black bone MRI sequences with the combined use of mandibular coils has enabled improved characterization and staging of benign and malignant changes of soft and hard tissues, such as accurate depth extension of clinically suspicious lesions and providing information on other clinically unsuspected changes [25].

A comparison of black bone MRI sequences in their application in the dental field confirmed that they are best suited to overcome the limitations of hard tissue imaging in oral and maxillofacial radiology [12,17,33]. The STIR sequence offered the best signal-to-noise ratio and contrast-to-noise ratio between nerves and muscles, while the DESS protocols were suitable for comparing quantitative parameters [17,33]. For visualization of more complex medical diseases, especially space-occupying malignant lesions, the contrast-enhanced 3D SPACE STIR sequence could accurately detect the localization of the pathological process with higher spatial resolution and image quality [34]. Combining a 14 + 1 receive coil array with black bone MRI sequences used in this setup provided three-dimensional, high-resolution dental and maxillomandibular imaging data with a better signal-to-noise ratio, faster imaging data acquisition by parallel imaging, and subsequent k-space undersampling and reduction of the occurrence of motion artifacts. It can provide highly accurate volumetric cross-sectional reconstructions of craniofacial structures, particularly soft-tissues such as peripheral nerves, mandibular canal, maxillary sinus, and periodontal ligaments, as well as tumor and tumor-like lesions, and minimize perioperative risks and complications by providing valuable additional information for the treating surgeon [25]. Therefore, after further validation of these data in larger cohorts, dental MRI using black bone MRI protocols has the potential to establish itself as an imaging modality in postoperative radiology workflows and provide postoperative care with an improved benefit–risk ratio. They can potentially lead to a higher detection rate of potential soft-tissue tumor recurrences, and thus improve patient outcomes. However, further studies should be performed to optimize the image quality with respect to the contrast-to-noise ratio of adjacent soft-tissues, as it appears relatively low for the evaluated DESS sequence.

Several guidelines should be considered before performing three-dimensional imaging procedures. First, cross-sectional imaging—despite being considered an indispensable tool in modern medical diagnosis—must always complement thorough clinical examination, rather than be a “stand-alone**”** diagnostic modality. Second, both conventional X-ray-based imaging and MRI can visualize anatomy and pathological changes. They complement each other with their different indications, strengths, and weaknesses. The clinician must decide which imaging modality is indicated respectively most appropriate from medical, ethical, economic, and patient-specific perspectives. The interpretation of imaging data should always consider the patient’s medical history, concomitant diseases, and previous interventions that may affect the imaged structures. From a clinical perspective, it is important to understand that obtaining a high standard of diagnostic information is a complex process that is always influenced by three fundamental factors—the patient, the imaging device, and the image detector.

As demonstrated in the case report of this article, the STIR imaging protocol in combination with the 15-channel mandibular coil is a further step towards personalized medicine and can be used for depicting soft-tissue pathologies. This additional information can provide useful supplemental information to assess what type of lesion is present and minimize the risks and ineffectiveness in preoperative decision-making by taking into account patient-specific factors. In conclusion, a thorough multidisciplinary coordinated preoperative evaluation of all case-specific factors, including medical history, clinical, and especially radiological and histopathological features, is crucial in borderline cases to provide the best possible treatment of oral cavity soft-tissue tumors.

## Figures and Tables

**Table 1:** A summary of the main technical parameters of the Black Bone MRI sequences applied in the study, namely the 3D Double-Echo Steady-State (DESS) and 3D Short-Tau Inversion Recovery (STIR) MRI protocols.

Black Bone MRI	3D Double-Echo Steady-State (DESS)	3D Short-Tau Inversion Recovery (STIR)
Acquisition time	12:24 min:s	7:04 min:s
FOV	242 × 242 × 78 mm^3^	210 × 242 × 78 mm^3^
Acquisition matrix	320 × 320 × 78	256 × 243 × 64
Acquisition voxel	0.75 × 0.75 × 0.75 mm^3^	0.82 × 0.86 × 1.0 mm^3^
Number of signal averages	1	1.4
TR	11.16 ms	3300 ms
TE1	4.21 ms	197 ms
TE2	7.7 ms	–
TI	–	220 ms
WFS (pix)/bandwidth (Hz)	1/355	1/630
Fat suppression	Selective water excitation	None

## Data Availability

The data presented in this study are available on request from the corresponding author. The data are not publicly available due to privacy restrictions.
